# Impact of Urate-Lowering Agents on Renal Outcomes in Chronic Kidney Disease: A Systematic Review

**DOI:** 10.7759/cureus.89343

**Published:** 2025-08-04

**Authors:** Hegwa Gafar Abubakir Osman, Ammar Abderahim, Maria Mohamed Ahmed Babiker, Mohammed Hassan Mohammed Saied, Eiman Elzein Abdelrahman Elsheikh, Alaa Elmutaz Mohamed Mahmoud, Hiba Karimeldin Mohamed Ali

**Affiliations:** 1 General Practice, Albadr Medical Center, Makkah, SAU; 2 Internal Medicine, Manchester Royal Infirmary Hospital, Manchester, GBR; 3 Internal Medicine, Medway Maritime Hospital, Kent, GBR; 4 General Medicine, Queen Elizabeth The Queen Mother Hospital, Kent, GBR; 5 General Internal Medicine, Medway Maritime NHS Foundation Trust, Kent, GBR; 6 Cardiology, Lincoln County Hospital, Lincoln, GBR; 7 Internal Medicine, Russells Hall Hospital, Dudley, GBR

**Keywords:** allopurinol, chronic kidney disease, febuxostat, hyperuricemia, renal outcomes, systematic review, urate-lowering agents

## Abstract

Chronic kidney disease (CKD) poses a significant global health burden, with hyperuricemia emerging as a potential modifiable risk factor for disease progression. Urate-lowering agents (ULAs) have been hypothesized to preserve renal function by reducing serum uric acid (SUA) levels and mitigating associated pathogenic mechanisms. However, clinical evidence regarding their efficacy remains inconsistent. This systematic review aimed to evaluate the effects of ULAs on renal outcomes in CKD patients by synthesizing evidence from recent placebo-controlled randomized trials. A comprehensive search of PubMed, Scopus, Web of Science, Embase, and ClinicalTrials.gov was conducted to identify randomized controlled trials (RCTs) published between 2020 and 2025. Ten studies met the inclusion criteria, assessing allopurinol, febuxostat, verinurad, and topiroxostat. The risk of bias was evaluated using the Cochrane Risk of Bias 2 (ROB 2) tool (London, United Kingdom). Data were synthesized narratively due to clinical and methodological heterogeneity. Febuxostat demonstrated potential renal benefits, with significant estimated glomerular filtration rate (eGFR) preservation in three studies. Allopurinol showed neutral effects on eGFR decline in large trials. Albuminuria reduction was observed with verinurad plus febuxostat but not with other ULAs. Safety profiles were favorable across studies, with no significant differences in adverse events versus placebo. While febuxostat may slow CKD progression in select populations, evidence for allopurinol and combination therapies remains inconclusive. Heterogeneity in outcomes underscores the need for personalized treatment and further research to identify optimal candidates for ULA therapy.

## Introduction and background

Chronic kidney disease (CKD) remains a significant global health burden, with increasing prevalence and substantial morbidity and mortality [1]. Hyperuricemia is frequently observed in patients with CKD, and there is growing interest in its potential role as not only a marker but also a contributor to disease progression [2]. Elevated serum uric acid (SUA) levels have been associated with endothelial dysfunction, increased oxidative stress, inflammation, and intrarenal crystal deposition, mechanisms that may accelerate kidney injury [3]. Consequently, urate-lowering agents (ULAs) have been hypothesized to slow CKD progression through the reduction of serum uric acid levels and the attenuation of these pathogenic pathways [4].

ULAs, particularly xanthine oxidase inhibitors, may exert renoprotective effects through multiple mechanisms. By lowering serum uric acid levels, they may reduce urate-induced oxidative stress and endothelial injury, which in turn improves renal hemodynamics and glomerular filtration [4]. Furthermore, ULAs have been shown to reduce intrarenal inflammation and fibrosis by inhibiting pro-inflammatory cytokines and suppressing the activation of the renin-angiotensin-aldosterone system (RAAS). Importantly, some clinical and experimental studies suggest that ULAs may reduce albuminuria and proteinuria, potentially by improving glomerular endothelial function and decreasing glomerular hypertension, both key contributors to protein leakage in CKD [5].

Among ULAs, xanthine oxidase inhibitors such as allopurinol and febuxostat have been most commonly studied, aiming to reduce uric acid production [5,6]. However, despite biological plausibility and supportive data from experimental models, clinical trial evidence assessing their efficacy in preserving kidney function has been inconsistent [7]. While some studies have reported beneficial effects on estimated glomerular filtration rate (eGFR) decline and albuminuria reduction [8], others have shown neutral results [9], leading to uncertainty regarding the therapeutic role of ULAs for renal protection in CKD.

Given these conflicting findings and the evolving evidence base, there is a need to systematically review and synthesize the most recent data to inform clinical practice. Previous meta-analyses have included studies with significant methodological heterogeneity and limited representation of contemporary trials. Therefore, this systematic review aimed to evaluate randomized placebo-controlled trials published in the last five years to determine whether urate-lowering therapy improves renal outcomes in CKD patients, focusing on the latest high-quality evidence to address this important clinical question.

## Review

Methods

Study Design

This study was conducted as a systematic review of randomized placebo-controlled trials, following the Preferred Reporting Items for Systematic Reviews and Meta-Analyses (PRISMA) 2020 guidelines, to evaluate the effects of urate-lowering agents on renal outcomes in patients with chronic kidney disease [10].

Eligibility Criteria

This systematic review included randomized placebo-controlled trials assessing the effects of urate-lowering agents on renal outcomes in patients with CKD. Studies published between January 2020 and June 2025 were included to capture the most recent and relevant evidence reflecting current clinical practices and pharmacological developments. Only studies involving adult participants with CKD, regardless of stage, were considered. Trials comparing any urate-lowering agent to a placebo were eligible. Observational studies, case reports, letters, editorials, reviews, and studies not reporting renal outcomes were excluded.

Information Sources

A comprehensive literature search was conducted across five electronic databases: PubMed, Scopus, Web of Science, Embase, and ClinicalTrials.gov. These databases were chosen to ensure wide coverage of biomedical and clinical trial literature, minimizing the risk of missing relevant studies.

Search Strategy

Search strategies were designed specifically for each database using a combination of Medical Subject Heading (MeSH) and free-text terms related to “urate-lowering agents,” “chronic kidney disease,” and “placebo-controlled trials.” The searches were restricted to articles published in English between 2020 and 2025. Reference lists of included studies and relevant reviews were also screened to identify any additional eligible studies.

Selection Process

All identified records were imported into EndNote (Clarivate, London, United Kingdom) for de-duplication. Two reviewers independently screened titles and abstracts to assess eligibility. Full texts of potentially relevant studies were retrieved and assessed in detail against the inclusion criteria. Disagreements were resolved through discussion and consensus, involving a third reviewer when necessary.

Data Collection Process

Data extraction was performed independently by two reviewers using a standardized data extraction form. Extracted data included study characteristics (author, year, country, study design, and sample size), participant characteristics (age, sex, and CKD stage), intervention details (urate-lowering agent type and dose), comparator details, the duration of follow-up, baseline serum uric acid levels, primary renal outcomes assessed (e.g., eGFR, albuminuria, and kidney function decline), effect sizes, confidence intervals (CI), p-values, and adverse events related to the intervention. Any discrepancies in data extraction were resolved by discussion.

Risk of Bias Assessment

The risk of bias for included randomized controlled trials (RCTs) was assessed independently by two reviewers using the Cochrane Risk of Bias 2 (ROB 2) tool (London, United Kingdom) [11]. This tool evaluates potential bias in five domains: randomization process, deviations from intended interventions, missing outcome data, the measurement of the outcome, and the selection of the reported result. Each domain was rated as low risk, some concerns, or high risk of bias. Disagreements were resolved through discussion.

Data Synthesis

Due to the heterogeneity of included studies in terms of patient populations, CKD stages, urate-lowering agents used, dosing regimens, and definitions of renal outcomes, a quantitative synthesis through meta-analysis or meta-regression was not performed. Additionally, variability in the reporting of effect measures and differences in follow-up durations across studies further limited the feasibility of meta-analysis. Therefore, results were synthesized narratively, presenting the findings from each included study descriptively and discussing consistencies and differences in reported outcomes.

Results

Study Selection

**Figure 1:**
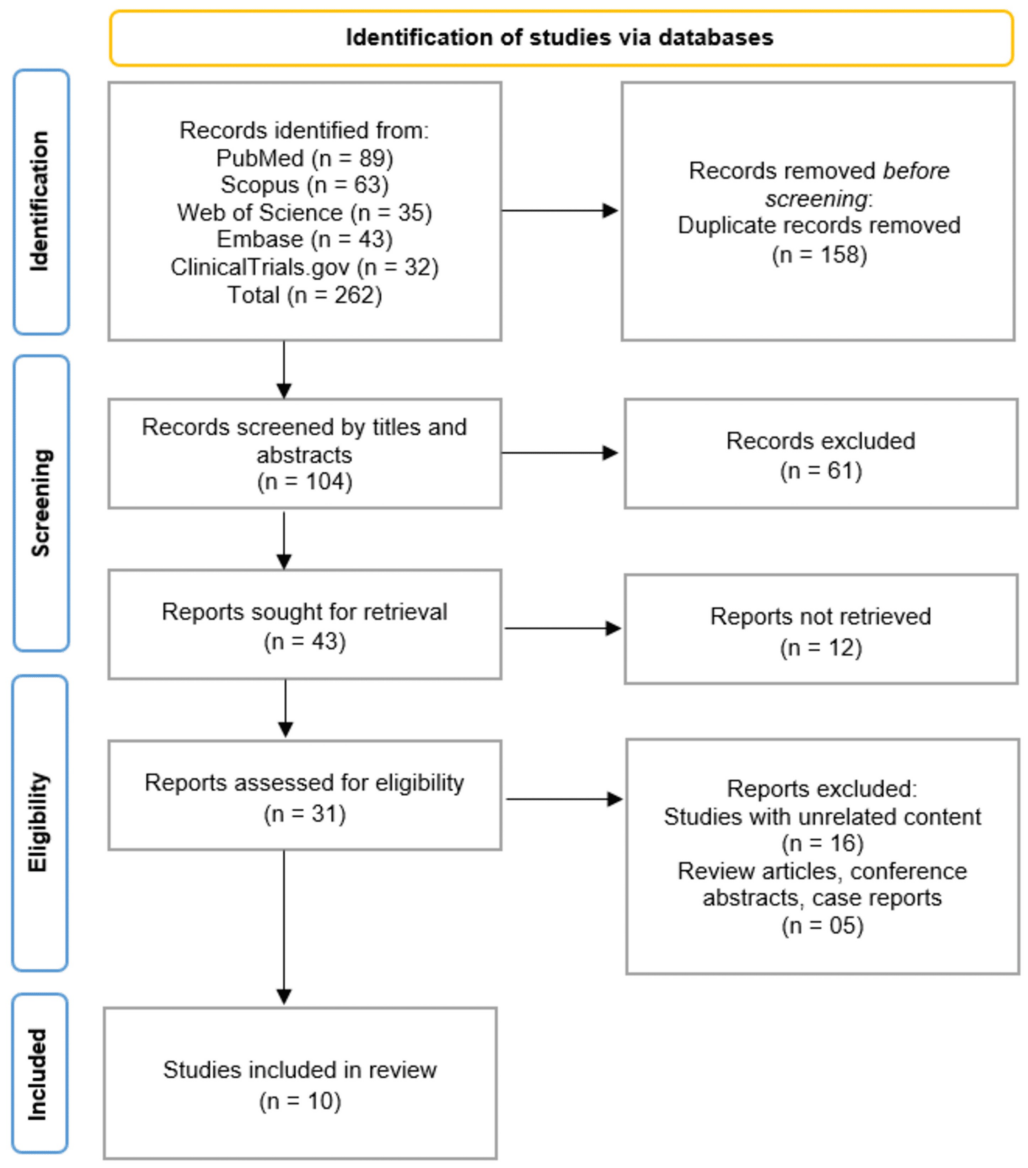
PRISMA Flow Diagram Illustrating the Study Selection Process PRISMA: Preferred Reporting Items for Systematic Reviews and Meta-Analyses

The study selection process followed the PRISMA guidelines and is summarized in the attached flowchart. A total of 262 records were initially identified through database searches, including PubMed (n = 89), Scopus (n = 63), Web of Science (n = 35), Embase (n = 43), and ClinicalTrials.gov (n = 32). After removing 158 duplicate records, 104 studies underwent title and abstract screening. Of these, 61 records were excluded for not meeting the inclusion criteria. The remaining 43 full-text articles were assessed for eligibility, with 12 reports unavailable for retrieval. After detailed evaluation, 21 studies were excluded for the following reasons: unrelated content (n = 16) and being review articles, conference abstracts, or case reports (n = 5). Ultimately, 10 studies met the eligibility criteria and were included in this systematic review (Figure 1) [12-21].

Characteristics of Included Studies

The systematic review included 10 RCTs evaluating the effects of ULAs on renal outcomes in patients with CKD [12-21]. The studies varied in design, sample size, population characteristics, and interventions (Table 1). Allopurinol was the most commonly studied ULA, investigated in five trials [12,13,15,16,21], while febuxostat was evaluated in four studies [14,17,18,20]. Other agents included verinurad (combined with febuxostat or allopurinol) [16,21] and topiroxostat [19]. Study durations ranged from eight weeks to three years, with sample sizes varying from 42 to 861 participants. Populations included patients with CKD stages 3-4, diabetic nephropathy, and asymptomatic hyperuricemia. SUA levels were generally elevated, with means ranging from 6.1 to 8.55 mg/dL.

**Table 1:** Studies’ Characteristics CKD, chronic kidney disease; CKD-EPI, Chronic Kidney Disease Epidemiology Collaboration; GFR, glomerular filtration rate; HbA1c, glycated hemoglobin; KIM-1, kidney injury molecule-1; NIDDK, National Institute of Diabetes and Digestive and Kidney Diseases; NGAL, neutrophil gelatinase-associated lipocalin; NR, not reported; RAAS, renin-angiotensin-aldosterone system; T2DM, type 2 diabetes mellitus; UMIN, University Hospital Medical Information Network; USA, United States of America; jRCT, Japan Registry of Clinical Trials; TGF-β1, transforming growth factor beta 1

Author (Year)	Study Location	Study Design	Sample Size	Population Characteristics	Intervention (Urate-Lowering Agent)	Comparator	Duration of Follow-Up	Baseline Serum Uric Acid (SUA) Level	Primary Renal Outcome(s)	Key Findings Related to Renal Outcomes
Badve et al. (2020) [12]	Australia and New Zealand	Randomized controlled trial	363 (182 allopurinol and 181 placebo)	Adults with stage 3 or 4 CKD, no gout history, mean eGFR of 31.7 mL/minute/1.73 m², and median urinary albumin-to-creatinine ratio (UACR) of 716.9 mg/g	Allopurinol (100-300 mg daily)	Placebo	104 weeks (~2 years)	Mean of 8.2 mg/dL	Change in estimated glomerular filtration rate (eGFR) from randomization to week 104	No significant difference in eGFR decline between allopurinol and placebo groups; mean difference of -0.10 mL/minute/1.73 m² per year (P = 0.85)
Doria et al. (2020) [13]	USA (multicenter trial funded by NIDDK)	Double-blind randomized controlled trial	530 (267 allopurinol and 263 placebo)	Adults with type 1 diabetes, mean age of 51.1 years, mean diabetes duration of 34.6 years, mean HbA1c of 8.2%, eGFR of 40-99.9 mL/minute/1.73 m², and evidence of diabetic kidney disease	Allopurinol	Placebo	3 years + 2-month washout	≥4.5 mg/dL (mean of 6.1 mg/dL)	Baseline-adjusted iohexol-based GFR after three years plus washout	No significant GFR difference; similar annual decline in both groups; allopurinol group had 40% higher urinary albumin excretion; no renal benefit observed
Nata et al. (2023) [14]	Thailand	Randomized controlled trial	84 (42 intervention and 42 control)	CKD stage 3-4 patients with asymptomatic hyperuricemia	Febuxostat 40 mg/day	Matching control	8 weeks	NR	Estimated glomerular filtration rate (eGFR) and albuminuria	Febuxostat lowered uric acid (UA) and preserved eGFR in patients with reduced uric acid (2.01 versus 0.04 mL/minute/1.73 m²; P = 0.030), with no overall eGFR or albuminuria difference
Perrenoud et al. (2020) [15]	USA	Double-blind randomized placebo-controlled trial (post hoc analysis)	80	Stage 3 CKD patients with asymptomatic hyperuricemia	Allopurinol 300 mg/day	Placebo	12 weeks	NR	Urinary biomarkers (albumin-to-creatinine ratio {ACR}, NGAL, KIM-1, and TGF-β1), CKD-EPI eGFR, and cystatin C (cys-C) eGFR	Allopurinol significantly lowered serum uric acid by -3.3 mg/dL but showed no significant improvement in urinary biomarkers of kidney damage or eGFR compared to placebo
Stack et al. (2021) [16]	Multicenter; funded by AstraZeneca	Phase 2, multicenter, prospective, randomized, double-blind, parallel-group, placebo-controlled trial	60	Adults of ≥18 years with hyperuricemia, T2DM, and albuminuria	Verinurad (9 mg) + febuxostat (80 mg)	Placebo	24 weeks	NR	Change in UACR from baseline after 12 weeks	Verinurad plus febuxostat reduced UACR by ~39% at 12 weeks and ~49% at 24 weeks compared to placebo; no meaningful change in eGFR or serum creatinine (SCr); well tolerated overall
Wen et al. (2020) [17]	China	Randomized controlled trial	42	CKD stage 3 diabetic nephropathy patients with high serum uric acid (≥360 µmol/L)	Febuxostat (20 mg) + low-purine diet + RAAS inhibitors + hypoglycemic treatment	Low-purine diet + RAAS inhibitors + hypoglycemic treatment (no febuxostat)	24 weeks	≥360 µmol/L	SUA, serum creatinine, cystatin C, eGFR, 24-hour urine protein, and ACR	Febuxostat reduced SUA, SCr, and cys-C and improved eGFR significantly versus control; also reduced tubular markers; effect on proteinuria and ACR significant after treatment; well tolerated but a short-term study
Yang et al. (2023) [18]	China	Prospective randomized controlled trial	100 (47 febuxostat and 45 control completed)	CKD stage 3-4 patients with asymptomatic hyperuricemia from seven medical centers	Febuxostat (dose titrated to achieve SUA of <6 mg/dL)	Control (no urate-lowering treatment)	12 months	NR	≥30% or 50% decline in eGFR, dialysis, or death from CKD at 12 months	Febuxostat group had significantly less eGFR decline (mean change +0.50 versus -4.46 mL/minute/1.73 m²; p = 0.006) and fewer reached primary outcomes compared to the control
Yamamoto et al. (2024) [19]	Japan (based on the UMIN/jRCT registry and topiroxostat approval region)	Multicenter randomized controlled trial	352	CKD patients with hyperuricemia	Topiroxostat (intensive therapy group targeting serum UA of ≥4.0 and <5.0 mg/dL)	Standard therapy group targeting serum UA of ≥6.0 and <7.0 mg/dL	52 weeks	Intensive: 8.15 mg/dL; standard: 8.23 mg/dL	Change in urine albumin-to-creatinine ratio (ACR) from baseline to week 52	No significant difference in log ACR change at 52 weeks between intensive and standard therapy groups
Azad et al. (2025) [20]	Bangladesh	Single-patient, blinded, placebo-controlled randomized trial	210 (105 in each group)	Patients with stage 3 and 4 CKD and asymptomatic hyperuricemia	Febuxostat 40 mg daily	Placebo	6 months	Febuxostat group: 8.55 mg/dL; placebo group: 8.10 mg/dL	Change in eGFR	Febuxostat lowered SUA from 8.55 to 4.92 mg/dL and increased eGFR from 25.28 to 27.01 mL/minute/1.73 m², while placebo saw an increase in SUA from 8.10 to 8.99 mg/dL and a decline in eGFR from 26.81 to 23.32 mL/minute/1.73 m²
Heerspink et al. (2024) [21]	China	Randomized placebo and active-controlled phase 2b trial	861	Adults with CKD, hyperuricemia (serum urate of ≥6.0 mg/dL), eGFR of ≥25 mL/minute/1.73 m², and UACR of 30-5000 mg/g; mean age of 65 years; 33% female; mean eGFR of 48 mL/minute/1.73 m²; median UACR of 217 mg/g	Verinurad (3 mg, 7.5 mg, and 12 mg) + allopurinol 300 mg/day	Placebo and placebo + allopurinol 300 mg/day	34 weeks (primary endpoint) and 60 weeks (secondary endpoint)	≥6.0 mg/dL	Change in urinary albumin-to-creatinine ratio (UACR) from baseline to 34 weeks	Verinurad + allopurinol further reduced serum urate dose dependently but did not decrease UACR or eGFR decline compared to allopurinol alone or placebo

Effects of Urate-Lowering Agents on Renal Function

The impact of ULAs on eGFR was mixed. Two large trials, Badve et al. [12] and Doria et al. [13], found no significant difference in eGFR decline between the allopurinol and placebo groups. Badve et al. [12] reported a mean difference of -0.10 mL/minute/1.73 m² per year (95% CI: -1.18 to +0.97; p = 0.85), while Doria et al. [13] observed an annual decline difference of -0.6 mL/minute/1.73 m² (p = 0.99). In contrast, febuxostat demonstrated favorable effects in several studies. Yang et al. [18] reported a significant between-group difference in eGFR change (+4.96 mL/minute/1.73 m²; p = 0.006) over 12 months, and Azad et al. [20] observed a 1.73 mL/minute/1.73 m² improvement in eGFR compared to placebo (p < 0.05). Nata et al. noted a subgroup benefit in eGFR (+1.97 mL/minute/1.73 m²; p = 0.030) among patients achieving reduced SUA levels [14].

Effects on Albuminuria and Proteinuria

Albuminuria outcomes were also heterogeneous. Stack et al. [16] reported a 39% reduction in UACR at 12 weeks with verinurad plus febuxostat (90% CI: -61.8% to -3.8%), while Wen et al. [17] found significant reductions in proteinuria markers with febuxostat (p < 0.05). Conversely, Yamamoto et al. observed no significant change in log ACR with intensive topiroxostat therapy (target SUA: 4.0-5.0 mg/dL) compared to standard therapy [19]. Similarly, Heerspink et al. found no UACR reduction with verinurad plus allopurinol versus placebo or allopurinol alone [21].

Biomarkers of Kidney Damage

Perrenoud et al. assessed urinary biomarkers (albumin-to-creatinine ratio {ACR}, neutrophil gelatinase-associated lipocalin {NGAL}, kidney injury molecule-1 {KIM-1}, and transforming growth factor beta 1 {TGF-β1}) and found no improvement despite a significant SUA reduction (-3.3 mg/dL; p < 0.001) [15]. Similarly, Doria et al. reported a 40% increase in urinary albumin excretion with allopurinol, suggesting no renal benefit [13].

Safety and Tolerability

ULAs were generally well tolerated. Serious adverse events were comparable between allopurinol and placebo in Badve et al. [12] (46% versus 44%) and Doria et al. [13]. Febuxostat studies reported no significant adverse events [14,17,18,20], and Stack et al. [16] noted good tolerability for verinurad plus febuxostat.

Summary of Key Findings

The evidence suggests that while febuxostat may preserve eGFR in specific CKD populations [14,18,20], allopurinol showed no consistent renal benefit [12,13,15]. Combined therapies (e.g., verinurad plus febuxostat) reduced albuminuria in some studies [16] but not others [21]. Heterogeneity in outcomes may reflect differences in study populations, follow-up durations, and SUA targets (Table 2).

**Table 2:** Renal Outcomes From Included Studies eGFR, estimated glomerular filtration rate; GFR, glomerular filtration rate; ADMA, asymmetric dimethylarginine; hs-CRP, high-sensitivity C-reactive protein; ACR, albumin-to-creatinine ratio; NGAL, neutrophil gelatinase-associated lipocalin; TGF-β1, transforming growth factor beta 1; KIM-1, kidney injury molecule-1; CKD-EPI, Chronic Kidney Disease Epidemiology Collaboration; GLMM, generalized linear mixed model; UACR, urinary albumin-to-creatinine ratio; SCr; serum creatinine; ULT, urate-lowering therapy

Author (Year)	Intervention Versus Comparator	Outcome Assessed	Effect Size	95% Confidence Interval (CI)	P-value	Direction of Effect	Adjustment for Confounders	Adverse Events Related to Intervention
Badve et al. (2020) [12]	Allopurinol versus placebo	Change in eGFR from randomization to week 104	-0.10 mL/minute/1.73 m² per year	-1.18 to +0.97	0.85	No significant difference	Not reported (NR)	Serious adverse events in 46% (allopurinol) versus 44% (placebo)
Doria et al. (2020) [13]	Allopurinol versus placebo	Baseline-adjusted iohexol-based GFR after three years + two-month washout: 0.001 mL/minute/1.73 m²; annual GFR decline: -0.6 mL/minute/1.73 m²/year; urinary albumin excretion rate: 40% higher with allopurinol	GFR difference: 0.001 mL/minute/1.73 m²; annual decline difference: -0.6 mL/minute/1.73 m²/year; albuminuria: +40%	GFR: -1.9 to 1.9; annual decline: -1.5 to 0.4; albuminuria: 0-80	GFR: 0.99; annual decline and albuminuria p-values not reported	No significant difference in GFR outcomes; albuminuria higher with allopurinol	Adjusted for baseline GFR	Similar frequency of serious adverse events in both groups
Nata et al. (2023) [14]	Febuxostat (40 mg/day) versus control	Serum uric acid (SUA) reduction; eGFR change (subgroup with decreased uric acid {UA}); serum ADMA; hs-CRP; albuminuria	-3.05 mg/dL (uric acid); +1.97 mL/minute/1.73 m² (eGFR subgroup); no significant difference (ADMA, hs-CRP, and albuminuria)	-3.43 to -2.67 (uric acid); 0.27-3.67 (eGFR subgroup)	<0.001 (uric acid); 0.030 (eGFR subgroup); NS (others)	Favors intervention for uric acid and eGFR (subgroup); no effect on other outcomes	NR	None observed
Perrenoud et al. (2020) [15]	Allopurinol versus placebo	ACR, NGAL, TGF-β1, KIM-1, and eGFR (CKD-EPI, cystatin C {cys-C})	No significant improvement; uric acid decreased to -3.3 mg/dL	-4.1 to -2.5 for uric acid	<0.001 for uric acid	decreased uric acid; no renal biomarker benefit	Adjusted (GLMM) Wilcoxon for KIM-1	NR
Stack et al. (2021) [16]	Verinurad (9 mg) + febuxostat (80 mg) versus placebo	UACR reduction at 12 weeks	-39.4%	90% CI: -61.8 to -3.8	NR	Reduced UACR	NR	Well tolerated; no clinically meaningful adverse events
Wen et al. (2020) [17]	Febuxostat + standard care versus standard care alone	SUA, SCr, cys-C, eGFR, 24-hour urine protein, albuminuria, and ACR	Significant difference between groups post treatment	NR	P < 0.05	Favors febuxostat (reduction in SUA, SCr, and cys-C; increase in eGFR; reduction in proteinuria markers)	NR	Well tolerated; no specific adverse events reported
Yang et al. (2023) [18]	Febuxostat versus control	Change in eGFR after 12 months	+4.96 mL/minute/1.73 m² (difference between groups)	NR	0.006	Favors febuxostat (slowed eGFR decline)	NR	No significant difference between groups
Yamamoto et al. (2024)[19]	Intensive ULT (topiroxostat; target UA of 4.0-5.0 mg/dL) versus standard ULT (target UA of 6.0-7.0 mg/dL)	Change in log urine ACR from baseline to 52 weeks	No significant difference reported	NR	Not reported (no significant difference)	No effect	NR	NR
Azad et al. (2025) [20]	Febuxostat (40 mg daily) versus placebo	eGFR change from baseline to six months	+1.73 mL/minute/1.73 m² increase in the febuxostat group versus -3.49 mL/minute/1.73 m² decrease in the placebo group	NR	<0.05	Favoring intervention (febuxostat improved or maintained eGFR compared to decline in placebo)	Diuretics, antihypertensives, and antidiabetics administered as necessary but no formal multivariable adjustment reported	NR
Heerspink et al. (2024) [21]	Verinurad (3 mg, 7.5 mg, and 12 mg) + allopurinol 300 mg/day versus placebo and allopurinol alone	Change in UACR at 34 weeks	3 mg, 16.7% reduction; 7.5 mg, 15.0% reduction; and 12 mg, 14.0% reduction, versus placebo: 37.3% reduction	3 mg, -0.6 to 37.1; 7.5 mg, -1.85 to 34.6; 12 mg, -3.4 to 34.4; placebo, 16.6-61.8	NR	No significant difference between groups	NR	Adverse events and serious adverse events were balanced among treatment groups

Risk of Bias Results

**Figure 2:**
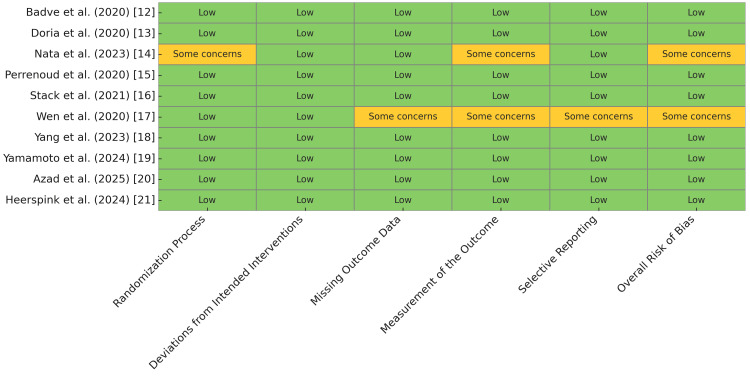
Risk of Bias Using the ROB 2 Tool ROB 2: Risk of Bias 2

The risk of bias assessment, conducted using the ROB 2 tool, revealed that most included studies demonstrated a low risk of bias across all domains, including the randomization process, deviations from intended interventions, missing outcome data, the measurement of the outcome, and selective reporting. Specifically, the studies by Badve et al. [12], Doria et al. [13], Perrenoud et al. [15], Stack et al. [16], Yang et al. [18], Yamamoto et al. [19], Azad et al. [20], and Heerspink et al. [21] were rated as having low overall risk of bias. However, some concerns were identified in two studies: Nata et al. [14] had some concerns due to issues in the randomization process and the measurement of the outcome, while Wen et al. [17] raised some concerns due to missing outcome data, the measurement of the outcome, and selective reporting (Figure 2).

Discussion

The findings of this systematic review provide a comprehensive evaluation of the effects of ULAs on renal outcomes in patients with CKD, synthesizing evidence from 10 RCTs [12-21]. The results reveal a complex and heterogeneous landscape, with some agents demonstrating potential benefits while others showed neutral or inconsistent effects. The most striking observation was the differential impact of ULAs on eGFR and albuminuria, which varied not only by drug class but also by patient population and study design. For instance, allopurinol, the most extensively studied ULA, failed to show consistent renal benefits across multiple large trials [12,13,15]. Badve et al. [12] and Doria et al. [13] reported no significant difference in eGFR decline between allopurinol and placebo, with the latter even noting a paradoxical 40% increase in urinary albumin excretion, suggesting that urate-lowering alone may not suffice to modify CKD progression in certain subgroups. These findings align with prior meta-analyses, such as those by Micanovic et al., which concluded that allopurinol’s renoprotective effects are marginal at best and may be context-dependent, particularly in non-gout populations [22].

In contrast, febuxostat emerged as a more promising candidate for renal protection, particularly in Asian populations with CKD stages 3-4 [14,17,18,20]. Yang et al. [18] reported a significant between-group difference in eGFR change (+4.96 mL/minute/1.73 m²; p = 0.006) over 12 months, while Azad et al. [20] observed a 1.73 mL/minute/1.73 m² improvement compared to placebo. These findings are supported by mechanistic studies suggesting that febuxostat, a selective xanthine oxidase inhibitor, may confer additional anti-inflammatory and endothelial benefits beyond urate-lowering, as highlighted by Nata et al. [14]. However, the generalizability of these results is limited by the predominance of single-center studies and the relatively short follow-up durations (e.g., eight weeks in Nata et al. [14]). Notably, the positive effects of febuxostat were not replicated in all trials, such as the neutral findings by Yamamoto et al., which may reflect differences in SUA targets or baseline patient characteristics [19]. This heterogeneity underscores the need for individualized treatment strategies, as recently emphasized in the KDIGO 2024 guidelines, which recommend considering ULAs only in hyperuricemic CKD patients with progressive disease despite the optimal management of traditional risk factors [23].

The impact of ULAs on albuminuria and proteinuria was equally variable. While Stack et al. [16] reported a 39% reduction in urinary albumin-to-creatinine ratio (UACR) with verinurad plus febuxostat, Heerspink et al. [21] found no significant UACR reduction with verinurad plus allopurinol. This discrepancy may be attributed to differences in drug mechanisms; verinurad, a urate transporter 1 (URAT1) inhibitor, may have distinct effects on the tubular handling of albumin compared to xanthine oxidase inhibitors. The albuminuria findings are particularly relevant given its established role as a surrogate marker for CKD progression, as demonstrated in the CREDENCE trial, which showed that interventions reducing albuminuria often correlate with slower eGFR decline [24]. However, the lack of consistent albuminuria reduction across studies [16,19,21] suggests that ULAs may not uniformly target the pathways driving proteinuric kidney disease, echoing the conclusions of a review [25] that questioned the routine use of ULAs for this indication. It is also possible that the variability in results is influenced by differences in patient populations, baseline proteinuria levels, concomitant medications (e.g., RAAS blockers or sodium-glucose cotransporter-2 {SGLT2} inhibitors), and study duration. Furthermore, while some preclinical studies have suggested anti-inflammatory and endothelial-protective effects of urate-lowering therapy that could theoretically reduce albuminuria, such mechanisms were not consistently explored or confirmed in the included trials [24]. This highlights the need for future studies to better characterize the subgroups of patients who may benefit from ULAs in terms of albuminuria reduction and to clarify the underlying mechanisms through mechanistic endpoints.

Safety and tolerability were generally favorable across studies, with no significant differences in serious adverse events between the ULA and placebo groups [12,13,16,20]. This is reassuring, particularly given the historical concerns about cardiovascular risks associated with febuxostat, as raised by the CARES trial [26]. However, the relatively short durations of most included trials (e.g., 12 weeks in Perrenoud et al. [15]) preclude definitive conclusions about long-term safety, a limitation also noted in the FDA’s 2019 advisory on febuxostat [27].

The risk of bias assessment revealed that most studies were methodologically robust, with low risk of bias across key domains [12,13,15,16,18-21]. However, some concerns were identified by Nata et al. [14] and Wen et al. [17], primarily due to issues in randomization and outcome measurement. These limitations highlight the need for larger, multicenter RCTs with standardized outcome assessments to minimize heterogeneity and strengthen causal inferences.

Limitations

This review has several limitations. First, the included studies exhibited significant clinical and methodological heterogeneity, particularly in terms of patient populations (e.g., diabetic versus non-diabetic CKD), follow-up durations, and SUA targets, which precluded meta-analysis. Second, most trials were underpowered to detect hard renal endpoints (e.g., dialysis initiation or mortality), relying instead on surrogate markers such as eGFR and albuminuria. Third, the predominance of industry-funded studies raises the possibility of reporting bias, as negative findings may be underrepresented [16,19,21].

## Conclusions

While febuxostat may offer renal benefits in specific CKD populations, the evidence for allopurinol and combination therapies remains inconclusive. The heterogeneity in outcomes underscores the importance of personalized treatment decisions, guided by patient-specific factors such as the etiology of CKD, baseline SUA levels, and comorbidities. Future research should prioritize long-term, event-driven RCTs to clarify the role of ULAs in CKD management and identify subgroups most likely to benefit. Until then, ULAs should be used judiciously, with the careful monitoring of renal parameters and adherence to current clinical guidelines.
